# Supplementary material for: Barnacles Mating Optimizer Algorithm to Extract the Parameters of the Photovoltaic Cells and Panels
Source: Sensors (Basel). 2022 Sep 15;22(18):6989. doi: 10.3390/s22186989 (PMC9504652; doi:10.3390/s22186989)
Supplement: Supplementary file 1 [file sensors-22-06989-s001.zip › sensors-1911554-supplementary.pdf]

Suplimentary files

Parameters obtained using different algorithms

Table S1. Monocrystalline silicon photovoltaic cell parameters (SDM and DDM)

Algorithm	I_{ph} [A]	I_{o1} [A]	n_1	R_s [Ω]	R_{sh} [Ω]	I_{o2} [A]	n_2
BMOA	4.2660E-01	5.4453E-08	1.4429	0.11186	50.305		
SDA	4.25752E-01	5.16853E-07	1.679294	0.091316	99.136671		
GA	4.25688E-01	8.38331E-07	1.73926	0.0859435	123.3659		
5P	4.255E-01	0.30645E-06	1.618311	0.10352224	145.222		
BMOA	4.2731E-01	5.6095E-08	1.4532	0.10822	53.106	5.3674E-07	2.0685
PSDA	4.25748E-01	2.40258E-06	1.6062857	0.0928610	103.5227	1.03013E-06	2.091868

Table S2. Amorphous silicon photovoltaic cell parameters (SDM and DDM)

Algorithm	I_{ph} [A]	I_{o1} [A]	n_1	R_s [Ω]	R_{sh} [Ω]	I_{o2} [A]	n_2
BMOA	1.1235E-02	1.7698E-06	3.7077E+00	0.0241	729.23		
SDA	1.1347E-02	0.70475E-06	3.353834	0.040283	520.065069		
5P	1.1247E-02	0.31475E-06	3.5658	0.03123	812.134		
BMOA	1.1389E-02	5.0617E-07	3.2431	0.1541	472.27	1.0000E-12	3.8000
PSDA	1.13491E-02	2.2219E-07	3.0794621	0.1987938	528.100826	1.06053E-06	4.09911

Table S3. RTC silicon photovoltaic cell parameters (SDM and DDM)

Algorithm	I_{ph} [A]	I_{o1} [A]	n_1	R_s [Ω]	R_{sh} [Ω]	I_{o2} [A]	n_2
BMOA	0.76441	6.3129E-06	1.8578E+00	1.9959E-02	100.00		
HSDA	0.7607758	0.32301E-06	1.48118232	0.03637708	53.71452		
PS	0.7617	0.9980E-06	1.6000	0.0313	64.1026		
ICA	0.7603	0.1465E-06	1.4421	0.0389	41.1577		
CPSO	0.7607	0.4000E-06	1.5033	0.0354	59.012		
MPCOA	0.7607	0.3366E-06	1.4817	0.0364	54.6328		
SA	0.762	0.4798E-06	1.5172	0.0345	43.1034		

FPA	0.76077	0.3106E-06	1.47707	0.03654	52.8771		
CS	0.7608	0.3230E-06	1.481	0.03634	53.7185		
EEGWO	0.732082	0.78558E-06	1.57598	0.034931	34.524598		
GA	0.7619	0.8087E-06	1.5751	0.0299	42.3729		
5P	0.7612	0.1966E-06	1.43	0.042	95.28		
BMOA	0.76263	1.0000E-12	2.0000	0.023399	99.937	3.5301E-06	1.7701
PSDA	0.7607941296	0.22670E-06	1.451	0.036762	55.398385	0.8267E-06	2.03036276
PS	0.7002	0.9889E-06	1.6	0.032	81.3008	0.0001E-06	1.981
ICA	0.7605	0.65637E-06	1.5970	0.0294	50.0000	1.5751E-10	1.0000
MPCOA	0.7608	0.3126E-06	1.4784	0.0364	54.2531	0.0453E-06	1.7846
SA	0.7623	0.4767E-06	1.5172	0.01	43.1034	0.0345E-06	2
FPA	0.76079	0.3008E-06	1.4747	0.0363	52.347	0.16615E-06	2
EEGWO	0.7256427	0.62356E-06	1.582721	0.016366	79.376488	0.51720E-06	1.784336

Table S4. PWP201 photovoltaic panel parameters (SDM and DDM)

Algorithm	I_{ph} [A]	I_{o1} [A]	n_1	R_s [Ω]	R_{sh} [Ω]	I_{o2} [A]	n_2
BMOA	1.0312	4.5218E-06	49.675	1.1683	999.78		
HSDA	1.0305143	0.34822E-06	48.642835	1.201271	981.98228037		
PS	1.0313	3.1756E-06	48.2889	1.2053	714.2857		
GCPSO	1.032382	2.51292E-06	49,4298	1.239288	744.716635		
WDOWOAPSO	1.03238234	2.5129E-06	49,4294	1.23928866	744.714358		
MPCOA	1.03188	3.3737E-06	48.50646	1.20295	849.6927		
SA	1.0331	3.6642E-06	48.8211	1.1989	833.3333		
FPA	1.032091	3.0475E-06	48.13128	1.217583	811.3721		
EEGWO	0.89531	2.7486E-06	48.3989	0.5654	642.53671		
5P	1.034	3.571E-06	48.71	1.206	1123.00		
BMOA	1.0411	1.0853E-06	44.566	1.3052	3.7451E+02	1.0000E-12	31.753

HSDA	1.032270	2.51196E-06	47.422967	1.2349375	737.27967	1.00838E-12	47.73212158
CPSO	1.03238233	2.51291639	47,4329674	1.2392884	744.71539	1.00005E−12	47,419837
TVACPSO	1.031434	2.63812E-06	47,555956	1.235632	821.65281	1E-12	100

Table S5. Sharp ND-R250A5 photovoltaic panel parameters (SDM and DDM)

Algorithm	I_{ph} [A]	I_{o1} [A]	n_1	R_s [Ω]	R_{sh} [Ω]	I_{o2} [A]	n_2
BMOA	9.2269	9.8302E-06	84.341	0.53520	5500		
HSDA	9.1435605	0.99811E-06	72.403381	0.59133379	5041.38450		
GCPSO	9.14486543	9.95854E-07	72,3947454	0.59187049	4999.9999		
5P	9.13242	9.452E-06	79.341	0.61220	4819.1		
BMOA	9.0690	1.0000E-12	49.966	0.66079	3029.1	1.3240E-08	57.132
HSDA	9.1449255	0.25699E-06	72.9078605	0.5894475	8346.5465	0.83734E-06	72.790804
GCPSO	9.1448653	2.16048E-07	72,3947478	0.59187053	4999.9999	7.798037E-7	72,394734

Table S6. Kyocera KC200GT photovoltaic panel parameters (SDM and DDM)

Algorithm	I_{ph} [A]	I_{o1} [A]	n_1	R_s [Ω]	R_{sh} [Ω]	I_{o2} [A]	n_2
BMOA	8.1400	7.6704E-06	92.687	0.049389	500.00		
HSDA	8.1861146	3.95444E-10	54.3328	0.26546856	125.588		
ICA	8.2100	0.09825E-06	70,2	0.00409	415.41		
WDO	8.1812	0.4423E-06	76,5288	0.1132	747.41		
BMOA	8.1362	1.4810E-06	98.760	0.042709	444.36	6.8870E-06	92.509
HSDA	8.199373	3.7243E-10	62.80799	0.28804863	110.0343	3.9374E-11	49.461
ICA	8.2100	0.14290E-06	71,6796	0.0038	108.784	0.02643E-10	58,5954

Results for the (I,V) points and parameters for the 6 photovoltaic cells and panels

Table S7 . (I, V) points of Monocrystalline silicon photovoltaic cell for 500 Epochs.

**Measured data**		**SDM-BMOA**	**DDM-BMOA**	**Error Values**	
**V [V]**	**I [A]**	**I_c [A]**	**I_c [A]**	**SDM**	**DDM**
0.0000E+00	4.2481E-01	4.2510E-01	4.2441E-01	2.8765E-04	-4.0167E-04

1.1822E-02	4.2480E-01	4.2489E-01	4.2426E-01	9.8917E-05	-5.3619E-04
2.0097E-02	4.2479E-01	4.2475E-01	4.2416E-01	-3.1303E-05	-6.2850E-04
5.3199E-02	4.2475E-01	4.2419E-01	4.2374E-01	-5.5644E-04	-1.0025E-03
9.8122E-02	4.2465E-01	4.2342E-01	4.2318E-01	-1.2286E-03	-1.4719E-03
1.1172E-01	4.2464E-01	4.2319E-01	4.2300E-01	-1.4558E-03	-1.6386E-03
1.4009E-01	4.2448E-01	4.2270E-01	4.2264E-01	-1.7811E-03	-1.8399E-03
2.0984E-01	4.2372E-01	4.2146E-01	4.2168E-01	-2.2523E-03	-2.0334E-03
2.6599E-01	4.2275E-01	4.2032E-01	4.2069E-01	-2.4365E-03	-2.0635E-03
3.0087E-01	4.2140E-01	4.1934E-01	4.1974E-01	-2.0521E-03	-1.6557E-03
3.2274E-01	4.2019E-01	4.1848E-01	4.1884E-01	-1.7104E-03	-1.3477E-03
3.5052E-01	4.1759E-01	4.1680E-01	4.1704E-01	-7.8791E-04	-5.4467E-04
3.9604E-01	4.0947E-01	4.1070E-01	4.1050E-01	1.2217E-03	1.0249E-03
4.1377E-01	0,403773	4.0596E-01	4.0551E-01	2.1892E-03	1.7374E-03
4.5396E-01	3.8056E-01	3.8436E-01	3.8330E-01	3.8040E-03	2.7451E-03
4.8175E-01	3.5006E-01	3.5361E-01	3.5238E-01	3.5528E-03	2.3225E-03
4.9357E-01	3.3110E-01	3.3470E-01	3.3354E-01	3.6046E-03	2.4386E-03
4.9534E-01	0,328027	3.3148E-01	3.3034E-01	3.4569E-03	2.3104E-03
5.1721E-01	2.8237E-01	2.8304E-01	2.8233E-01	6.7151E-04	-3.4978E-05
0.531988	2.4221E-01	2.4071E-01	2.4045E-01	-1.4994E-03	-1.7622E-03
5.3672E-01	2.2709E-01	2.2584E-01	2.2570E-01	-1.2474E-03	-1.3840E-03
5.3908E-01	2.1931E-01	2.1807E-01	2.1800E-01	-1.2316E-03	-1.3054E-03
5.5918E-01	1.4596E-01	1.4392E-01	1.4423E-01	-2.0390E-03	-1.7324E-03
5.6391E-01	1.2639E-01	1.2483E-01	1.2513E-01	-1.5610E-03	-1.2623E-03
5.7396E-01	8.2714E-02	8.1808E-02	8.1940E-02	-9.0575E-04	-7.7401E-04
5.7928E-01	5.7940E-02	5.8304E-02	5.8211E-02	3.6397E-04	2.7133E-04
5.8223E-01	4.4104E-02	4.4628E-02	4.4390E-02	5.2406E-04	2.8566E-04
5.8873E-01	1.2269E-02	1.4343E-02	1.3641E-02	2.0743E-03	1.3722E-03
5.9121E-01	0.0000E+00	2.3484E-03	1.4381E-03	2.3484E-03	1.4381E-03

Table S8. Monocrystalline silicon photovoltaic cell parameters and range for 500 epochs

Algorithm	I_{ph} [A]	I_{o1} [A]	n_1	R_s [Ω]	R_{sh} [Ω]	I_{o2} [A]	n_2
Range Set SMD	0-1	E-12 – E-5	1-2	0-1	0-200		
BMOA SMD	4.2591E-01	5.7106E-08	1.4471E+00	1.1222E-01	5.8617E+01		
Range Set DDM	0-1	E-12– E-5	1-2	0-1	0-200	E-12 – E-5	1-3
BMOA DDM	4.2498E-01	5.6103E-08	1.4534E+00	1.0842E-01	8.0337E+01	5.1887E-07	2.0471E+00

Table S9. (I, V) points of Monocrystalline silicon photovoltaic cell for 30 populations.

Measured data		SDM-BMOA	DDM-BMOA	Error Values	
V [V]	I [A]	I_c [A]	I_c [A]	SDM	DDM
0.0000E+00	4.2481E-01	4.2508E-01	4.3611E-01	2.7382E-04	1.1299E-02
1.1822E-02	4.2480E-01	4.2500E-01	4.3499E-01	2.0287E-04	1.0199E-02
2.0097E-02	4.2479E-01	4.2494E-01	4.3422E-01	1.5502E-04	9.4307E-03
5.3199E-02	4.2475E-01	4.2470E-01	4.3110E-01	-4.1580E-05	6.3523E-03
9.8122E-02	4.2465E-01	4.2438E-01	4.2686E-01	-2.7258E-04	2.2100E-03
1.1172E-01	4.2464E-01	4.2427E-01	4.2557E-01	-3.6815E-04	9.3071E-04
1.4009E-01	4.2448E-01	4.2406E-01	4.2288E-01	-4.2430E-04	-1.5952E-03
2.0984E-01	4.2372E-01	4.2341E-01	4.1618E-01	-3.0393E-04	-7.5316E-03
2.6599E-01	4.2275E-01	4.2254E-01	4.1052E-01	-2.1661E-04	-1.2236E-02
3.0087E-01	4.2140E-01	4.2150E-01	4.0665E-01	1.0276E-04	-1.4743E-02
3.2274E-01	4.2019E-01	4.2043E-01	4.0396E-01	2.3774E-04	-1.6235E-02
3.5052E-01	4.1759E-01	4.1821E-01	4.0000E-01	6.2282E-04	-1.7593E-02
3.9604E-01	4.0947E-01	4.1032E-01	3.9097E-01	8.4223E-04	-1.8503E-02
4.1377E-01	0,403773	4.0457E-01	3.8590E-01	7.9442E-04	-1.7872E-02
4.5396E-01	3.8056E-01	3.8060E-01	3.6795E-01	4.1324E-05	-1.2608E-02
4.8175E-01	3.5006E-01	3.4932E-01	3.4612E-01	-7.3625E-04	-3.9380E-03
4.9357E-01	3.3110E-01	3.3079E-01	3.3293E-01	-3.1310E-04	1.8346E-03
4.9534E-01	0,328027	3.2767E-01	3.3069E-01	-3.5945E-04	2.6662E-03

5.1721E-01	2.8237E-01	2.8136E-01	2.9587E-01	-1.0066E-03	1.3507E-02
0.531988	2.4221E-01	2.4112E-01	2.6255E-01	-1.0921E-03	2.0337E-02
5.3672E-01	2.2709E-01	2.2681E-01	2.4969E-01	-2.7276E-04	2.2607E-02
5.3908E-01	2.1931E-01	2.1933E-01	2.4281E-01	2.5700E-05	2.3502E-02
5.5918E-01	1.4596E-01	1.4682E-01	1.6931E-01	8.5938E-04	2.3355E-02
5.6391E-01	1.2639E-01	1.2762E-01	1.4735E-01	1.2249E-03	2.0963E-02
5.7396E-01	8.2714E-02	8.3604E-02	9.3227E-02	8.8982E-04	1.0513E-02
5.7928E-01	5.7940E-02	5.8864E-02	5.9873E-02	9.2421E-04	1.9334E-03
5.8223E-01	4.4104E-02	4.4404E-02	3.9759E-02	3.0035E-04	-4.3449E-03
5.8873E-01	1.2269E-02	1.1628E-02	-8.9198E-03	-6.4080E-04	-2.1189E-02
5.9121E-01	0.0000E+00	-1.4743E-03	-2.9192E-02	-1.4743E-03	-2.9192E-02

Table S10 . Monocrystalline silicon photovoltaic cell parameters and range for 30 populations

Algorithm	I_{ph} [A]	I_{o1} [A]	n_1	R_s [Ω]	R_{sh} [Ω]	I_{o2} [A]	n_2
Range Set SMD	0-1	E-12 – E-5	1-2	0-1	0-200		
BMOA SMD	4.2534E-01	7.7851E-07	1.7310E+00	8.7058E-02	1.4172E+02		
Range Set DDM	0-1	E-12– E-5	1-2	0-1	0-200	E-12 – E-5	1-3
BMOA DDM	4.3611E-01	2.5937E-06	1.9096E+00	0.0000E+00	1.0628E+01	1.0000E-12	2.5909E+00

Table S11. (I, V) points of Monocrystalline silicon photovoltaic cell for 15 populations.

Measured data		SDM-BMOA	DDM-BMOA	Error Values	
V [V]	I [A]	I_c [A]	I_c [A]	SDM	DDM
0.0000E+00	4.2481E-01	4.7520E-01	4.2650E-01	5.0389E-02	1.6867E-03
1.1822E-02	4.2480E-01	4.6944E-01	4.2643E-01	4.4649E-02	1.6371E-03
2.0097E-02	4.2479E-01	4.6542E-01	4.2639E-01	4.0633E-02	1.6038E-03
5.3199E-02	4.2475E-01	4.4931E-01	4.2621E-01	2.4563E-02	1.4618E-03
9.8122E-02	4.2465E-01	4.2745E-01	4.2594E-01	2.7963E-03	1.2895E-03
1.1172E-01	4.2464E-01	4.2083E-01	4.2585E-01	-3.8144E-03	1.2061E-03

1.4009E-01	4.2448E-01	4.0702E-01	4.2564E-01	-1.7459E-02	1.1627E-03
2.0984E-01	4.2372E-01	3.7308E-01	4.2490E-01	-5.0640E-02	1.1791E-03
2.6599E-01	4.2275E-01	3.4575E-01	4.2363E-01	-7.7005E-02	8.8279E-04
3.0087E-01	4.2140E-01	3.2877E-01	4.2210E-01	-9.2622E-02	6.9874E-04
3.2274E-01	4.2019E-01	3.1813E-01	4.2056E-01	-1.0206E-01	3.6333E-04
3.5052E-01	4.1759E-01	3.0461E-01	4.1753E-01	-1.1298E-01	-6.2146E-05
3.9604E-01	4.0947E-01	2.8246E-01	4.0779E-01	-1.2702E-01	-1.6803E-03
4.1377E-01	0,403773	2.7382E-01	4.0125E-01	-1.2995E-01	-2.5233E-03
4.5396E-01	3.8056E-01	2.5423E-01	3.7604E-01	-1.2633E-01	-4.5156E-03
4.8175E-01	3.5006E-01	2.4063E-01	3.4528E-01	-1.0944E-01	-4.7847E-03
4.9357E-01	3.3110E-01	2.3480E-01	3.2746E-01	-9.6298E-02	-3.6342E-03
4.9534E-01	0,328027	2.3392E-01	3.2449E-01	-9.4103E-02	-3.5351E-03
5.1721E-01	2.8237E-01	2.2300E-01	2.8059E-01	-5.9362E-02	-1.7794E-03
0.531988	2.4221E-01	2.1544E-01	2.4231E-01	-2.6769E-02	9.5525E-05
5.3672E-01	2.2709E-01	2.1297E-01	2.2851E-01	-1.4119E-02	1.4225E-03
5.3908E-01	2.1931E-01	2.1172E-01	2.2127E-01	-7.5865E-03	1.9676E-03
5.5918E-01	1.4596E-01	2.0061E-01	1.5006E-01	5.4654E-02	4.0990E-03
5.6391E-01	1.2639E-01	1.9782E-01	1.3071E-01	7.1431E-02	4.3173E-03
5.7396E-01	8.2714E-02	1.9153E-01	8.5698E-02	1.0882E-01	2.9839E-03
5.7928E-01	5.7940E-02	1.8796E-01	5.9805E-02	1.3002E-01	1.8652E-03
5.8223E-01	4.4104E-02	1.8587E-01	4.4606E-02	1.4177E-01	5.0157E-04
5.8873E-01	1.2269E-02	1.8100E-01	9.5252E-03	1.6873E-01	-2.7438E-03
5.9121E-01	0.0000E+00	1.7902E-01	-4.6081E-03	1.7902E-01	-4.6081E-03

Table S12. Monocrystalline silicon photovoltaic cell parameters and range for 15 populations

Algorithm	I_{ph} [A]	I_{o1} [A]	n_1	R_s [Ω]	R_{sh} [Ω]	I_{o2} [A]	n_2
Range Set SMD	0-1	E-12 – E-5	1-2	0-1	0-200		

BMOA SMD	4.7520E-01	1.0000E-12	1.0000E+00	0.0000E+00	2.0548E+00		
Range Set DDM	0-1	E-12– E-5	1-2	0-1	0-200	E-12 – E-5	1-3
BMOA DDM	4.2663E-01	4.4316E-06	1.9975E+00	5.8995E-02	2.0000E+02	7.3554E-06	3.0000E+00

Table S13. (I, V) points of aSi photovoltaic cell for 500 Epochs.

Measured data		SDM-BMOA	DDM-BMOA	Error Values	
V [V]	I [A]	I_c [A]	I_c [A]	SDM	DDM
-1.0000E-02	1.1283E-02	1.1332E-02	1.1428E-02	4.9126E-05	1.4472E-04
8.4800E-03	1.1266E-02	1.1303E-02	1.1388E-02	3.7727E-05	1.2195E-04
2.6955E-02	1.1243E-02	1.1274E-02	1.1347E-02	3.1860E-05	1.0478E-04
4.5435E-02	1.1224E-02	1.1245E-02	1.1307E-02	2.1692E-05	8.3353E-05
6.3915E-02	1.1202E-02	1.1216E-02	1.1267E-02	1.3714E-05	6.4188E-05
8.2390E-02	1.1193E-02	1.1187E-02	1.1226E-02	-6.2929E-06	3.3081E-05
1.0087E-01	1.1155E-02	1.1157E-02	1.1186E-02	2.6284E-06	3.0996E-05
1.1935E-01	1.1110E-02	1.1128E-02	1.1145E-02	1.7250E-05	3.4729E-05
1.3783E-01	1.1099E-02	1.1098E-02	1.1104E-02	-1.2612E-06	5.4699E-06
1.5631E-01	1.1078E-02	1.1068E-02	1.1064E-02	-1.0674E-05	-1.4529E-05
1.7479E-01	1.1040E-02	1.1037E-02	1.1023E-02	-2.7417E-06	-1.6989E-05
1.9326E-01	1.1007E-02	1.1006E-02	1.0982E-02	-1.0320E-06	-2.5439E-05
2.1175E-01	1.0982E-02	1.0974E-02	1.0940E-02	-7.1702E-06	-4.1473E-05
2.3020E-01	1.0941E-02	1.0942E-02	1.0898E-02	1.0277E-06	-4.2831E-05
2.4870E-01	1.0896E-02	1.0909E-02	1.0856E-02	1.3170E-05	-3.9892E-05
2.6715E-01	1.0861E-02	1.0876E-02	1.0814E-02	1.4572E-05	-4.7228E-05
2.8565E-01	1.0838E-02	1.0841E-02	1.0771E-02	3.0514E-06	-6.6989E-05
3.0415E-01	1.0777E-02	1.0804E-02	1.0726E-02	2.6859E-05	-5.0814E-05
3.2260E-01	1.0724E-02	1.0766E-02	1.0682E-02	4.2707E-05	-4.1870E-05
3.4110E-01	1.0690E-02	1.0726E-02	1.0635E-02	3.6537E-05	-5.4137E-05

3.5955E-01	1.0624E-02	1.0684E-02	1.0588E-02	6.0038E-05	-3.5760E-05
3.7805E-01	1.0596E-02	1.0638E-02	1.0538E-02	4.2362E-05	-5.7467E-05
3.9650E-01	1.0523E-02	1.0589E-02	1.0487E-02	6.6725E-05	-3.5850E-05
4.1500E-01	1.0467E-02	1.0536E-02	1.0432E-02	6.8799E-05	-3.5064E-05
4.3350E-01	1.0407E-02	1.0477E-02	1.0374E-02	6.9883E-05	-3.3596E-05
4.5195E-01	1.0327E-02	1.0412E-02	1.0311E-02	8.5036E-05	-1.6169E-05
4.7045E-01	1.0258E-02	1.0339E-02	1.0243E-02	8.1845E-05	-1.4944E-05
4.8890E-01	1.0173E-02	1.0257E-02	1.0167E-02	8.4757E-05	-5.2497E-06
5.0740E-01	1.0060E-02	1.0164E-02	1.0083E-02	1.0372E-04	2.3156E-05
5.2585E-01	9.9670E-03	1.0056E-02	9.9880E-03	8.9235E-05	2.0952E-05
5.4435E-01	9.8534E-03	9.9319E-03	9.8790E-03	7.8462E-05	2.5592E-05
5.6285E-01	9.7021E-03	9.7871E-03	9.7529E-03	8.4997E-05	5.0802E-05
5.8130E-01	9.5669E-03	9.6181E-03	9.6058E-03	5.1197E-05	3.8946E-05
5.9980E-01	9.3838E-03	9.4187E-03	9.4317E-03	3.4893E-05	4.7911E-05
6.1825E-01	9.2011E-03	9.1836E-03	9.2248E-03	-1.7480E-05	2.3723E-05
6.3675E-01	8.9748E-03	8.9039E-03	8.9758E-03	-7.0917E-05	9.6915E-07
6.5520E-01	8.6853E-03	8.5717E-03	8.6756E-03	-1.1363E-04	-9.7197E-06
6.7370E-01	8.3376E-03	8.1738E-03	8.3097E-03	-1.6378E-04	-2.7899E-05
6.9215E-01	7.9110E-03	7.6988E-03	7.8640E-03	-2.1222E-04	-4.6977E-05
7.1065E-01	7.3725E-03	7.1273E-03	7.3160E-03	-2.4521E-04	-5.6529E-05
7.2915E-01	6.6650E-03	6.4402E-03	6.6414E-03	-2.2477E-04	-2.3598E-05
7.4760E-01	5.7870E-03	5.6155E-03	5.8113E-03	-1.7154E-04	2.4295E-05
7.6610E-01	4.7014E-03	4.6188E-03	4.7819E-03	-8.2645E-05	8.0483E-05
7.8455E-01	3.4034E-03	3.4195E-03	3.5099E-03	1.6143E-05	1.0647E-04
8.0305E-01	1.8769E-03	1.9676E-03	1.9271E-03	9.0674E-05	5.0220E-05
8.2150E-01	9.5797E-05	2.1784E-04	-3.3997E-05	1.2204E-04	-1.2979E-04

Table S14. Amorphous silicon photovoltaic cell parameters and range for 500 epochs

Algorithm	I_{ph} [A]	I_{o1} [A]	n_1	R_s [Ω]	R_{sh} [Ω]	I_{o2} [A]	n_2
Range Set SDM	0-0.1	E-12 – E-05	1-5	0-0.5	0-1000		
BMOA SDM	1.1317E-02	1.8668E-06	3.7313E+00	0.0000E+00	6.4748E+02		
Range Set DDM	0-0.1	E-12 – E-05	1-5	0-0.5	0-1000	E-12 – E-05	1-5
BMOA DDM	1.1406E-02	4.9562E-07	3.2372E+00	0.0000E+00	4.6025E+02	1.0000E-12	2.3300E+00

Table S15. (I, V) points of amorphous silicon photovoltaic cell for 30 populations.

Measured data		SDM-BMOA	DDM-BMOA	Error Values	
V [V]	I [A]	I_c [A]	I_c [A]	SDM	DDM
-1.0000E-02	1.1283E-02	1.1303E-02	1.1254E-02	1.9618E-05	-2.8963E-05
8.4800E-03	1.1266E-02	1.1274E-02	1.1230E-02	8.0888E-06	-3.6082E-05
2.6955E-02	1.1243E-02	1.1245E-02	1.1205E-02	2.1036E-06	-3.7724E-05
4.5435E-02	1.1224E-02	1.1215E-02	1.1180E-02	-8.1675E-06	-4.3725E-05
6.3915E-02	1.1202E-02	1.1186E-02	1.1155E-02	-1.6233E-05	-4.7604E-05
8.2390E-02	1.1193E-02	1.1157E-02	1.1130E-02	-3.6307E-05	-6.3585E-05
1.0087E-01	1.1155E-02	1.1127E-02	1.1104E-02	-2.7430E-05	-5.0718E-05
1.1935E-01	1.1110E-02	1.1098E-02	1.1078E-02	-1.2824E-05	-3.2240E-05
1.3783E-01	1.1099E-02	1.1068E-02	1.1052E-02	-3.1320E-05	-4.6995E-05
1.5631E-01	1.1078E-02	1.1038E-02	1.1025E-02	-4.0680E-05	-5.2757E-05
1.7479E-01	1.1040E-02	1.1007E-02	1.0998E-02	-3.2650E-05	-4.1291E-05
1.9326E-01	1.1007E-02	1.0976E-02	1.0971E-02	-3.0792E-05	-3.6173E-05
2.1175E-01	1.0982E-02	1.0945E-02	1.0943E-02	-3.6721E-05	-3.9035E-05
2.3020E-01	1.0941E-02	1.0913E-02	1.0914E-02	-2.8244E-05	-2.7710E-05
2.4870E-01	1.0896E-02	1.0880E-02	1.0884E-02	-1.5741E-05	-1.2583E-05
2.6715E-01	1.0861E-02	1.0847E-02	1.0853E-02	-1.3885E-05	-8.3602E-06
2.8565E-01	1.0838E-02	1.0813E-02	1.0820E-02	-2.4840E-05	-1.7212E-05
3.0415E-01	1.0777E-02	1.0777E-02	1.0786E-02	-3.4017E-07	9.1016E-06

3.2260E-01	1.0724E-02	1.0740E-02	1.0751E-02	1.6343E-05	2.7290E-05
3.4110E-01	1.0690E-02	1.0701E-02	1.0713E-02	1.1178E-05	2.3315E-05
3.5955E-01	1.0624E-02	1.0660E-02	1.0673E-02	3.5873E-05	4.8865E-05
3.7805E-01	1.0596E-02	1.0616E-02	1.0629E-02	1.9610E-05	3.3122E-05
3.9650E-01	1.0523E-02	1.0568E-02	1.0582E-02	4.5630E-05	5.9323E-05
4.1500E-01	1.0467E-02	1.0517E-02	1.0530E-02	4.9645E-05	6.3186E-05
4.3350E-01	1.0407E-02	1.0460E-02	1.0473E-02	5.2984E-05	6.6056E-05
4.5195E-01	1.0327E-02	1.0398E-02	1.0410E-02	7.0733E-05	8.3051E-05
4.7045E-01	1.0258E-02	1.0328E-02	1.0339E-02	7.0531E-05	8.1845E-05
4.8890E-01	1.0173E-02	1.0249E-02	1.0259E-02	7.6845E-05	8.6968E-05
5.0740E-01	1.0060E-02	1.0159E-02	1.0168E-02	9.9680E-05	1.0849E-04
5.2585E-01	9.9670E-03	1.0057E-02	1.0064E-02	8.9530E-05	9.7014E-05
5.4435E-01	9.8534E-03	9.9370E-03	9.9433E-03	8.3613E-05	8.9855E-05
5.6285E-01	9.7021E-03	9.7976E-03	9.8028E-03	9.5515E-05	1.0074E-04
5.8130E-01	9.5669E-03	9.6345E-03	9.6390E-03	6.7562E-05	7.2134E-05
5.9980E-01	9.3838E-03	9.4414E-03	9.4458E-03	5.7574E-05	6.2008E-05
6.1825E-01	9.2011E-03	9.2130E-03	9.2179E-03	1.1862E-05	1.6815E-05
6.3675E-01	8.9748E-03	8.9401E-03	8.9464E-03	-3.4675E-05	-2.8433E-05
6.5520E-01	8.6853E-03	8.6148E-03	8.6231E-03	-7.0516E-05	-6.2175E-05
6.7370E-01	8.3376E-03	8.2235E-03	8.2347E-03	-1.1411E-04	-1.0291E-04
6.9215E-01	7.9110E-03	7.7542E-03	7.7687E-03	-1.5682E-04	-1.4228E-04
7.1065E-01	7.3725E-03	7.1869E-03	7.2048E-03	-1.8555E-04	-1.6771E-04
7.2915E-01	6.6650E-03	6.5018E-03	6.5219E-03	-1.6324E-04	-1.4313E-04
7.4760E-01	5.7870E-03	5.6752E-03	5.6950E-03	-1.1177E-04	-9.2029E-05
7.6610E-01	4.7014E-03	4.6715E-03	4.6856E-03	-2.9943E-05	-1.5754E-05
7.8455E-01	3.4034E-03	3.4577E-03	3.4574E-03	5.4316E-05	5.3984E-05
8.0305E-01	1.8769E-03	1.9808E-03	1.9512E-03	1.0385E-04	7.4315E-05

8.2150E-01	9.5797E-05	1.9187E-04	1.1050E-04	9.6077E-05	1.4700E-05

Table S16. Amorphous silicon photovoltaic cell parameters and range for 30 populations

Algorithm	I_{ph} [A]	I_{o1} [A]	n_1	R_s [Ω]	R_{sh} [Ω]	I_{o2} [A]	n_2
Range Set SDM	0-0.1	E-12 – E-05	1-5	0-0.5	0-1000		
BMOA SDM	1.1287E-02	1.4860E-06	3.6351E+00	0.0000E+00	6.4307E+02		
Range Set DDM	0-0.1	E-12 – E-05	1-5	0-0.5	0-1000	E-12 – E-05	1-5
BMOA DDM	1.1241E-02	2.0573E-07	3.1051E+00	0.0000E+00	7.7467E+02	4.7616E-06	4.7532E+00

Table S17. (I, V) points of amorphous silicon photovoltaic cell for 15 populations.

Measured data		SDM-BMOA	DDM-BMOA	Error Values	
V [V]	I [A]	I_c [A]	I_c [A]	SDM	DDM
-1.0000E-02	1.1283E-02	1.1370E-02	1.1349E-02	8.7016E-05	6.6293E-05
8.4800E-03	1.1266E-02	1.1334E-02	1.1316E-02	6.8305E-05	4.9908E-05
2.6955E-02	1.1243E-02	1.1298E-02	1.1282E-02	5.5174E-05	3.9090E-05
4.5435E-02	1.1224E-02	1.1261E-02	1.1248E-02	3.7790E-05	2.4001E-05
6.3915E-02	1.1202E-02	1.1225E-02	1.1214E-02	2.2657E-05	1.1142E-05
8.2390E-02	1.1193E-02	1.1189E-02	1.1179E-02	-4.4348E-06	-1.3707E-05
1.0087E-01	1.1155E-02	1.1152E-02	1.1145E-02	-2.5018E-06	-9.5463E-06
1.1935E-01	1.1110E-02	1.1116E-02	1.1111E-02	5.2366E-06	3.8725E-07
1.3783E-01	1.1099E-02	1.1079E-02	1.1076E-02	-2.0050E-05	-2.2764E-05
1.5631E-01	1.1078E-02	1.1042E-02	1.1041E-02	-3.6094E-05	-3.6715E-05
1.7479E-01	1.1040E-02	1.1005E-02	1.1007E-02	-3.4616E-05	-3.3196E-05
1.9326E-01	1.1007E-02	1.0968E-02	1.0971E-02	-3.9163E-05	-3.5775E-05
2.1175E-01	1.0982E-02	1.0930E-02	1.0936E-02	-5.1333E-05	-4.6060E-05
2.3020E-01	1.0941E-02	1.0892E-02	1.0899E-02	-4.8873E-05	-4.1808E-05
2.4870E-01	1.0896E-02	1.0854E-02	1.0863E-02	-4.2160E-05	-3.3407E-05
2.6715E-01	1.0861E-02	1.0815E-02	1.0825E-02	-4.5802E-05	-3.5503E-05
2.8565E-01	1.0838E-02	1.0776E-02	1.0787E-02	-6.1946E-05	-5.0254E-05

3.0415E-01	1.0777E-02	1.0735E-02	1.0748E-02	-4.2232E-05	-2.9304E-05
3.2260E-01	1.0724E-02	1.0694E-02	1.0708E-02	-2.9877E-05	-1.5922E-05
3.4110E-01	1.0690E-02	1.0651E-02	1.0665E-02	-3.8871E-05	-2.4132E-05
3.5955E-01	1.0624E-02	1.0606E-02	1.0622E-02	-1.7379E-05	-2.1021E-06
3.7805E-01	1.0596E-02	1.0560E-02	1.0575E-02	-3.6179E-05	-2.0692E-05
3.9650E-01	1.0523E-02	1.0511E-02	1.0526E-02	-1.1871E-05	3.5216E-06
4.1500E-01	1.0467E-02	1.0458E-02	1.0473E-02	-8.6770E-06	6.2303E-06
4.3350E-01	1.0407E-02	1.0402E-02	1.0416E-02	-5.1270E-06	8.8816E-06
4.5195E-01	1.0327E-02	1.0341E-02	1.0354E-02	1.4003E-05	2.6685E-05
4.7045E-01	1.0258E-02	1.0274E-02	1.0285E-02	1.6469E-05	2.7329E-05
4.8890E-01	1.0173E-02	1.0199E-02	1.0208E-02	2.6881E-05	3.5439E-05
5.0740E-01	1.0060E-02	1.0115E-02	1.0121E-02	5.5410E-05	6.1191E-05
5.2585E-01	9.9670E-03	1.0020E-02	1.0022E-02	5.2586E-05	5.5081E-05
5.4435E-01	9.8534E-03	9.9092E-03	9.9080E-03	5.5795E-05	5.4581E-05
5.6285E-01	9.7021E-03	9.7808E-03	9.7756E-03	7.8679E-05	7.3474E-05
5.8130E-01	9.5669E-03	9.6303E-03	9.6209E-03	6.3408E-05	5.3970E-05
5.9980E-01	9.3838E-03	9.4516E-03	9.4381E-03	6.7845E-05	5.4266E-05
6.1825E-01	9.2011E-03	9.2389E-03	9.2215E-03	3.7834E-05	2.0374E-05
6.3675E-01	8.9748E-03	8.9828E-03	8.9621E-03	7.9563E-06	-1.2653E-05
6.5520E-01	8.6853E-03	8.6743E-03	8.6518E-03	-1.1041E-05	-3.3505E-05
6.7370E-01	8.3376E-03	8.2989E-03	8.2764E-03	-3.8663E-05	-6.1215E-05
6.9215E-01	7.9110E-03	7.8431E-03	7.8229E-03	-6.7869E-05	-8.8142E-05
7.1065E-01	7.3725E-03	7.2850E-03	7.2699E-03	-8.7500E-05	-1.0256E-04
7.2915E-01	6.6650E-03	6.6020E-03	6.5957E-03	-6.2976E-05	-6.9339E-05
7.4760E-01	5.7870E-03	5.7672E-03	5.7723E-03	-1.9772E-05	-1.4687E-05
7.6610E-01	4.7014E-03	4.7402E-03	4.7576E-03	3.8843E-05	5.6223E-05
7.8455E-01	3.4034E-03	3.4826E-03	3.5078E-03	7.9150E-05	1.0445E-04

8.0305E-01	1.8769E-03	1.9330E-03	1.9514E-03	5.6122E-05	7.4541E-05
8.2150E-01	9.5797E-05	3.4321E-05	1.2756E-05	-6.1476E-05	-8.3042E-05

.

Table S18. Amorphous silicon photovoltaic cell parameters and range for 15 populations

Algorithm	I_{ph} [A]	I_{o1} [A]	n_1	R_s [Ω]	R_{sh} [Ω]	I_{o2} [A]	n_2
Range Set SDM	0-0.1	E-12 – E-05	1-5	0-0.5	0-1000		
BMOA SDM	1.1355E-02	6.0364E-07	3.3010E+00	2.2123E-01	5.1214E+02		
Range Set DDM	0-0.1	E-12 – E-05	1-5	0-0.5	0-1000	E-12 – E-05	1-5
BMOA DDM	1.1341E-02	1.0000E-12	1.5503E+00	5.0000E-01	5.4850E+02	9.6014E-07	3.4995E+00

Table S19. (I, V) points of RTC silicon photovoltaic cell for 500 epochs.

Measured data		SDM-BMOA	DDM-BMOA	Error Values	
**V [V]**	**I [A]**	**I_c [A]**	**I_c [A]**	**SDM**	**DDM**
−0.2057	7.6400E-01	7.6603E-01	7.6525E-01	2.0269E-03	1.2528E-03
−0.1291	7.6200E-01	7.6525E-01	7.6449E-01	3.2542E-03	2.4867E-03
−0.0588	7.6050E-01	7.6454E-01	7.6378E-01	4.0434E-03	3.2826E-03
5.7000E-03	7.6050E-01	7.6389E-01	7.6313E-01	3.3857E-03	2.6329E-03
6.4600E-02	7.6000E-01	7.6327E-01	7.6253E-01	3.2691E-03	2.5287E-03
1.1850E-01	7.5900E-01	7.6266E-01	7.6194E-01	3.6607E-03	2.9440E-03
1.6780E-01	7.5700E-01	7.6200E-01	7.6133E-01	4.9957E-03	4.3266E-03
2.1320E-01	7.5700E-01	7.6113E-01	7.6056E-01	4.1337E-03	3.5586E-03
2.5450E-01	7.5550E-01	7.5983E-01	7.5943E-01	4.3349E-03	3.9303E-03
2.9240E-01	7.5400E-01	7.5765E-01	7.5753E-01	3.6479E-03	3.5295E-03
3.2690E-01	7.5050E-01	7.5389E-01	7.5420E-01	3.3879E-03	3.7036E-03
3.5850E-01	7.4650E-01	7.4747E-01	7.4839E-01	9.7376E-04	1.8868E-03
3.8730E-01	7.3850E-01	7.3699E-01	7.3863E-01	-1.5081E-03	1.3183E-04

4.1370E-01	7.2800E-01	7.2047E-01	7.2287E-01	-7.5293E-03	-5.1316E-03
4.3730E-01	7.0650E-01	6.9651E-01	6.9953E-01	-9.9867E-03	-6.9703E-03
4.5900E-01	6.7550E-01	6.6247E-01	6.6577E-01	-1.3031E-02	-9.7291E-03
4.7840E-01	6.3200E-01	6.1782E-01	6.2090E-01	-1.4184E-02	-1.1102E-02
4.9600E-01	5.7300E-01	5.6143E-01	5.6375E-01	-1.1571E-02	-9.2531E-03
5.1190E-01	4.9900E-01	4.9370E-01	4.9481E-01	-5.3042E-03	-4.1910E-03
5.2650E-01	4.1300E-01	4.1382E-01	4.1340E-01	8.1643E-04	4.0483E-04
5.3980E-01	3.1650E-01	3.2352E-01	3.2159E-01	7.0240E-03	5.0906E-03
5.5210E-01	2.1200E-01	2.2245E-01	2.1923E-01	1.0452E-02	7.2318E-03
5.6330E-01	1.0350E-01	1.1363E-01	1.0967E-01	1.0129E-02	6.1678E-03
5.7360E-01	−0.0100	-1.6276E-03	-5.3669E-03	8.3724E-03	4.6331E-03
5.8330E-01	−0.1230	-1.2675E-01	-1.2956E-01	-3.7482E-03	-6.5620E-03
5.9000E-01	−0.2100	-2.2147E-01	-2.2254E-01	-1.1473E-02	-1.2542E-02

Table S20. RTC silicon photovoltaic cell parameters and range for 500 epochs

Algorithm	I_{ph} [A]	I_{o1} [A]	n_1	R_s [Ω]	R_{sh} [Ω]	I_{o2} [A]	n_2
Range Set SDM	0–1	E-12 – E-05	1-2	0-0.5	0-100		
BMOA SDM	7.6410E-01	6.4273E-06	1.8608E+00	1.9664E-02	9.9147E+01		
Range Set DDM	0–1	E-12 – E-05	1-2	0-0.5	0-100	E-12 – E-05	1–2.5
BMOA DDM	7.6337E-01	1.0000E-12	2.0000E+00	2.3443E-02	9.9964E+01	3.6268E-06	1.7732E+00

Table S21. (I, V) points of RTC silicon photovoltaic cell for 30 populations.

Measured data		SDM-BMOA	DDM-BMOA	Error Values	
V [V]	I [A]	I_c [A]	I_c [A]	SDM	DDM
−0.2057	7.6400E-01	7.6338E-01	7.6422E-01	-6.1819E-04	2.2361E-04
−0.1291	7.6200E-01	7.6262E-01	7.6346E-01	6.1629E-04	1.4579E-03

−0.0588	7.6050E-01	7.6191E-01	7.6275E-01	1.4132E-03	2.2542E-03
5.7000E-03	7.6050E-01	7.6127E-01	7.6211E-01	7.6602E-04	1.6053E-03
6.4600E-02	7.6000E-01	7.6067E-01	7.6150E-01	6.6906E-04	1.5035E-03
1.1850E-01	7.5900E-01	7.6010E-01	7.6093E-01	1.1037E-03	1.9264E-03
1.6780E-01	7.5700E-01	7.5953E-01	7.6033E-01	2.5326E-03	3.3295E-03
2.1320E-01	7.5700E-01	7.5887E-01	7.5961E-01	1.8658E-03	2.6108E-03
2.5450E-01	7.5550E-01	7.5794E-01	7.5859E-01	2.4353E-03	3.0873E-03
2.9240E-01	7.5400E-01	7.5639E-01	7.5689E-01	2.3867E-03	2.8857E-03
3.2690E-01	7.5050E-01	7.5362E-01	7.5390E-01	3.1228E-03	3.3952E-03
3.5850E-01	7.4650E-01	7.4862E-01	7.4858E-01	2.1154E-03	2.0824E-03
3.8730E-01	7.3850E-01	7.3989E-01	7.3949E-01	1.3889E-03	9.9065E-04
4.1370E-01	7.2800E-01	7.2525E-01	7.2447E-01	-2.7535E-03	-3.5328E-03
4.3730E-01	7.0650E-01	7.0288E-01	7.0178E-01	-3.6250E-03	-4.7202E-03
4.5900E-01	6.7550E-01	6.6964E-01	6.6837E-01	-5.8601E-03	-7.1305E-03
4.7840E-01	6.3200E-01	6.2458E-01	6.2334E-01	-7.4198E-03	-8.6555E-03
4.9600E-01	5.7300E-01	5.6649E-01	5.6551E-01	-6.5117E-03	-7.4949E-03
5.1190E-01	4.9900E-01	4.9606E-01	4.9549E-01	-2.9394E-03	-3.5054E-03
5.2650E-01	4.1300E-01	4.1287E-01	4.1282E-01	-1.2935E-04	-1.8318E-04
5.3980E-01	3.1650E-01	3.1955E-01	3.1997E-01	3.0519E-03	3.4714E-03
5.5210E-01	2.1200E-01	2.1641E-01	2.1716E-01	4.4080E-03	5.1588E-03
5.6330E-01	1.0350E-01	1.0729E-01	1.0812E-01	3.7901E-03	4.6151E-03
5.7360E-01	−0.0100	-5.3833E-03	-4.8710E-03	4.6167E-03	5.1290E-03
5.8330E-01	−0.1230	-1.2569E-01	-1.2583E-01	-2.6947E-03	-2.8257E-03
5.9000E-01	−0.2100	-2.1389E-01	-2.1486E-01	-3.8884E-03	-4.8642E-03

Table S22. RTC silicon photovoltaic cell parameters and range for 30 populations

Algorithm	I_{ph} [A]	I_{o1} [A]	n_1	R_s [Ω]	R_{sh} [Ω]	I_{o2} [A]	n_2

Range Set SDM	0–1	E-12 – E-05	1-2	0-0.5	0-100		
BMOA SDM	7.6155E-01	1.4968E-06	1.6539E+00	2.9207E-02	1.0000E+02		
Range Set DDM	0–1	E-12 – E-05	1-2	0-0.5	0-100	E-12 – E-05	1–2.5
BMOA DDM	7.6238E-01	2.1925E-06	1.9452E+00	2.8308E-02	1.0000E+02	8.6733E-07	1.6150E+00

Table S23. (I, V) points of RTC silicon photovoltaic cell for 15 populations.

Measured data		SDM-BMOA	DDM-BMOA	Error Values	
V [V]	I [A]	I_c [A]	I_c [A]	SDM	DDM
−0.2057	7.6400E-01	7.7627E-01	7.5997E-01	1.2269E-02	-4.0277E-03
−0.1291	7.6200E-01	7.7165E-01	7.5920E-01	9.6509E-03	-2.7952E-03
−0.0588	7.6050E-01	7.6741E-01	7.5850E-01	6.9106E-03	-2.0031E-03
5.7000E-03	7.6050E-01	7.6351E-01	7.5784E-01	3.0143E-03	-2.6634E-03
6.4600E-02	7.6000E-01	7.5994E-01	7.5720E-01	-6.0521E-05	-2.7951E-03
1.1850E-01	7.5900E-01	7.5662E-01	7.5656E-01	-2.3754E-03	-2.4428E-03
1.6780E-01	7.5700E-01	7.5349E-01	7.5581E-01	-3.5104E-03	-1.1895E-03
2.1320E-01	7.5700E-01	7.5038E-01	7.5480E-01	-6.6230E-03	-2.1972E-03
2.5450E-01	7.5550E-01	7.4710E-01	7.5328E-01	-8.4044E-03	-2.2206E-03
2.9240E-01	7.5400E-01	7.4324E-01	7.5080E-01	-1.0755E-02	-3.2009E-03
3.2690E-01	7.5050E-01	7.3829E-01	7.4674E-01	-1.2212E-02	-3.7555E-03
3.5850E-01	7.4650E-01	7.3138E-01	7.4020E-01	-1.5123E-02	-6.2985E-03
3.8730E-01	7.3850E-01	7.2144E-01	7.3004E-01	-1.7059E-02	-8.4634E-03
4.1370E-01	7.2800E-01	7.0700E-01	7.1474E-01	-2.1001E-02	-1.3261E-02
4.3730E-01	7.0650E-01	6.8691E-01	6.9323E-01	-1.9588E-02	-1.3273E-02
4.5900E-01	6.7550E-01	6.5895E-01	6.6332E-01	-1.6555E-02	-1.2175E-02
4.7840E-01	6.3200E-01	6.2225E-01	6.2439E-01	-9.7541E-03	-7.6132E-03
4.9600E-01	5.7300E-01	5.7499E-01	5.7478E-01	1.9949E-03	1.7761E-03
5.1190E-01	4.9900E-01	5.1624E-01	5.1379E-01	1.7237E-02	1.4789E-02
5.2650E-01	4.1300E-01	4.4394E-01	4.3962E-01	3.0944E-02	2.6622E-02

5.3980E-01	3.1650E-01	3.5780E-01	3.5224E-01	4.1295E-02	3.5740E-02
5.5210E-01	2.1200E-01	2.5578E-01	2.4988E-01	4.3782E-02	3.7885E-02
5.6330E-01	1.0350E-01	1.3919E-01	1.3409E-01	3.5689E-02	3.0595E-02
5.7360E-01	−0.0100	7.1386E-03	4.2006E-03	1.7139E-02	1.4201E-02
5.8330E-01	−0.1230	-1.4385E-01	-1.4299E-01	-2.0847E-02	-1.9987E-02
5.9000E-01	−0.2100	-2.6606E-01	-2.6125E-01	-5.6057E-02	-5.1246E-02

Table S24. RTC silicon photovoltaic cell parameters and range for 15 populations

Algorithm	I_{ph} [A]	I_{o1} [A]	n_1	R_s [Ω]	R_{sh} [Ω]	I_{o2} [A]	n_2
Range Set SDM	0–1	E-12 – E-05	1-2	0-0.5	0-100		
BMOA SDM	7.6386E-01	1.0000E-05	1.9434E+00	0.0000E+00	1.6588E+01		
Range Set DDM	0–1	E-12 – E-05	1-2	0-0.5	0-100	E-12 – E-05	1–2.5
BMOA DDM	7.5790E-01	1.0000E-05	2.0000E+00	0.0000E+00	1.0000E+02	1.0000E-05	2.1728E+00

Table S25. (I, V) points of the PWP201 photovoltaic panel for 500 epochs.

Measured data		SDM-BMOA	DDM-BMOA	Error Values	
**V [V]**	**I [A]**	**I_c [A]**	**I_c [A]**	**SDM**	**DDM**
1.2480E-01	1.0315E+00	1.0307E+00	1.0398E+00	-7.8534E-04	8.3027E-03
1.8093E+00	1.0300E+00	1.0288E+00	1.0344E+00	-1.2162E-03	4.4239E-03
3.3511E+00	1.0260E+00	1.0270E+00	1.0295E+00	9.6170E-04	3.4857E-03
4.7622E+00	1.0220E+00	1.0251E+00	1.0249E+00	3.1458E-03	2.8928E-03
6.0538E+00	1.0180E+00	1.0231E+00	1.0205E+00	5.1382E-03	2.4957E-03
7.2364E+00	1.0155E+00	1.0206E+00	1.0160E+00	5.0569E-03	5.0226E-04
8.3189E+00	1.0140E+00	1.0167E+00	1.0109E+00	2.7231E-03	-3.1225E-03
9.3097E+00	1.0100E+00	1.0105E+00	1.0042E+00	5.3344E-04	-5.8107E-03
1.0216E+01	1.0035E+00	1.0003E+00	9.9438E-01	-3.2042E-03	-9.1239E-03
1.1045E+01	9.8800E-01	9.8384E-01	9.7928E-01	-4.1604E-03	-8.7230E-03
1.1802E+01	9.6300E-01	9.5851E-01	9.5603E-01	-4.4868E-03	-6.9663E-03
1.2493E+01	9.2550E-01	9.2171E-01	9.2161E-01	-3.7877E-03	-3.8926E-03

1.3123E+01	8.7250E-01	8.7162E-01	8.7363E-01	-8.7656E-04	1.1271E-03
1.3698E+01	8.0750E-01	8.0678E-01	8.1006E-01	-7.1863E-04	2.5578E-03
1.4222E+01	7.2650E-01	7.2858E-01	7.3210E-01	2.0764E-03	5.5970E-03
1.4700E+01	6.3450E-01	6.3826E-01	6.4100E-01	3.7614E-03	6.4978E-03
1.5135E+01	5.3450E-01	5.3822E-01	5.3942E-01	3.7161E-03	4.9248E-03
1.5531E+01	4.2750E-01	4.3217E-01	4.3174E-01	4.6697E-03	4.2378E-03
1.5893E+01	3.1850E-01	3.2179E-01	3.1988E-01	3.2871E-03	1.3832E-03
1.6223E+01	2.0850E-01	2.1032E-01	2.0765E-01	1.8164E-03	-8.4610E-04
1.6524E+01	1.0100E-01	9.8593E-02	9.5907E-02	-2.4075E-03	-5.0933E-03
1.6799E+01	−0.0080	-7.0927E-03	-7.9246E-03	9.0731E-04	7.5372E-05
1.7050E+01	−0.1110	-1.1124E-01	-1.0948E-01	-2.4014E-04	1.5171E-03
1.7279E+01	−0.2090	-2.1146E-01	-2.0614E-01	-2.4641E-03	2.8597E-03
1.7489E+01	−0.3030	-3.0540E-01	-2.9529E-01	-2.3980E-03	7.7122E-03

Table S26. PWP201 photovoltaic panel parameters and range for 500 epochs

Algorithm	I_{ph} [A]	I_{o1} [A]	n_1	R_s [Ω]	R_{sh} [Ω]	I_{o2} [A]	n_2
Range Set SDM	0–2	E-12 – E-05	1-2*36	0-2	0-1000		
BMOA SDM	1.0322E+00	4.4199E-06	4.9588E+01	1.1577E+00	8.8458E+02		
Range Set DDM	0–2	E-12 – E-05	1-2*36	0-2	0-1000	E-12 – E-05	1-2*36
BMOA DDM	1.0445E+00	1.0803E-06	4.4577E+01	1.2940E+00	3.1343E+02	1.0000E-12	3.1967E+01

Table 27. (I, V) points of the PWP201 photovoltaic panel for 30 populations.

Measured data		SDM-BMOA	DDM-BMOA	Error Values	
**V [V]**	**I [A]**	**I_c [A]**	**I_c [A]**	**SDM**	**DDM**
1.2480E-01	1.0315E+00	1.0323E+00	1.0303E+00	8.1804E-04	-1.1688E-03
1.8093E+00	1.0300E+00	1.0306E+00	1.0286E+00	5.9163E-04	-1.3826E-03
3.3511E+00	1.0260E+00	1.0289E+00	1.0270E+00	2.9326E-03	9.8933E-04
4.7622E+00	1.0220E+00	1.0272E+00	1.0253E+00	5.2167E-03	3.3416E-03
6.0538E+00	1.0180E+00	1.0252E+00	1.0235E+00	7.2083E-03	5.4695E-03

7.2364E+00	1.0155E+00	1.0225E+00	1.0210E+00	6.9701E-03	5.4790E-03
8.3189E+00	1.0140E+00	1.0183E+00	1.0172E+00	4.2557E-03	3.1738E-03
9.3097E+00	1.0100E+00	1.0114E+00	1.0109E+00	1.3988E-03	9.2861E-04
1.0216E+01	1.0035E+00	1.0002E+00	1.0005E+00	-3.3202E-03	-2.9695E-03
1.1045E+01	9.8800E-01	9.8248E-01	9.8380E-01	-5.5238E-03	-4.2016E-03
1.1802E+01	9.6300E-01	9.5578E-01	9.5809E-01	-7.2212E-03	-4.9133E-03
1.2493E+01	9.2550E-01	9.1771E-01	9.2082E-01	-7.7865E-03	-4.6776E-03
1.3123E+01	8.7250E-01	8.6672E-01	8.7025E-01	-5.7812E-03	-2.2519E-03
1.3698E+01	8.0750E-01	8.0156E-01	8.0496E-01	-5.9382E-03	-2.5388E-03
1.4222E+01	7.2650E-01	7.2366E-01	7.2642E-01	-2.8371E-03	-7.7874E-05
1.4700E+01	6.3450E-01	6.3423E-01	6.3593E-01	-2.6788E-04	1.4281E-03
1.5135E+01	5.3450E-01	5.3549E-01	5.3588E-01	9.8593E-04	1.3847E-03
1.5531E+01	4.2750E-01	4.3084E-01	4.3002E-01	3.3412E-03	2.5225E-03
1.5893E+01	3.1850E-01	3.2183E-01	3.2000E-01	3.3303E-03	1.4953E-03
1.6223E+01	2.0850E-01	2.1144E-01	2.0902E-01	2.9414E-03	5.2483E-04
1.6524E+01	1.0100E-01	1.0050E-01	9.7925E-02	-5.0313E-04	-3.0746E-03
1.6799E+01	−0.0080	-5.2588E-03	-7.0735E-03	2.7412E-03	9.2652E-04
1.7050E+01	−0.1110	-1.0980E-01	-1.1046E-01	1.2037E-03	5.3981E-04
1.7279E+01	−0.2090	-2.1086E-01	-2.0988E-01	-1.8579E-03	-8.8160E-04
1.7489E+01	−0.3030	-3.0621E-01	-3.0301E-01	-3.2075E-03	-7.5602E-06

Table S28. PWP201 photovoltaic panel parameters and range for 30 populations

Algorithm	I_{ph} [A]	I_{o1} [A]	n_1	R_s [Ω]	R_{sh} [Ω]	I_{o2} [A]	n_2
Range Set SDM	0–2	E-12 – E-05	1-2*36	0-2	0-1000		
BMOA SDM	1.0336E+00	8.0998E-06	5.2140E+01	1.0890E+00	1.0000E+03		

Range Set DDM	0–2	E-12 – E-05	1-2*36	0-2	0-1000	E-12 – E-05	1-2*36
BMOA DDM	1.0317E+00	1.0000E-12	7.2000E+01	1.1572E+00	1.0000E+03	4.9290E-06	5.0024E+01

Table S29. (I, V) points of the PWP201 photovoltaic panel for 15 populations.

Measured data		SDM-BMOA	DDM-BMOA	Error Values	
V [V]	I [A]	I_c [A]	I_c [A]	SDM	DDM
1.2480E-01	1.0315E+00	1.0323E+00	1.0323E+00	7.5771E-04	7.6729E-04
1.8093E+00	1.0300E+00	1.0305E+00	1.0291E+00	5.3181E-04	-8.5092E-04
3.3511E+00	1.0260E+00	1.0289E+00	1.0262E+00	2.8740E-03	2.4580E-04
4.7622E+00	1.0220E+00	1.0272E+00	1.0235E+00	5.1609E-03	1.4631E-03
6.0538E+00	1.0180E+00	1.0252E+00	1.0206E+00	7.1577E-03	2.6393E-03
7.2364E+00	1.0155E+00	1.0224E+00	1.0175E+00	6.9290E-03	1.9632E-03
8.3189E+00	1.0140E+00	1.0182E+00	1.0134E+00	4.2298E-03	-6.2982E-04
9.3097E+00	1.0100E+00	1.0114E+00	1.0074E+00	1.3953E-03	-2.6052E-03
1.0216E+01	1.0035E+00	1.0002E+00	9.9795E-01	-3.2942E-03	-5.5477E-03
1.1045E+01	9.8800E-01	9.8254E-01	9.8287E-01	-5.4637E-03	-5.1268E-03
1.1802E+01	9.6300E-01	9.5587E-01	9.5932E-01	-7.1275E-03	-3.6813E-03
1.2493E+01	9.2550E-01	9.1783E-01	9.2430E-01	-7.6669E-03	-1.2031E-03
1.3123E+01	8.7250E-01	8.6685E-01	8.7551E-01	-5.6504E-03	3.0141E-03
1.3698E+01	8.0750E-01	8.0168E-01	8.1101E-01	-5.8167E-03	3.5111E-03
1.4222E+01	7.2650E-01	7.2376E-01	7.3210E-01	-2.7436E-03	5.5985E-03
1.4700E+01	6.3450E-01	6.3428E-01	6.4010E-01	-2.1787E-04	5.6016E-03
1.5135E+01	5.3450E-01	5.3548E-01	5.3775E-01	9.8389E-04	3.2477E-03
1.5531E+01	4.2750E-01	4.3079E-01	4.2946E-01	3.2897E-03	1.9550E-03
1.5893E+01	3.1850E-01	3.2174E-01	3.1717E-01	3.2362E-03	-1.3264E-03
1.6223E+01	2.0850E-01	2.1132E-01	2.0471E-01	2.8202E-03	-3.7873E-03
1.6524E+01	1.0100E-01	1.0036E-01	9.2904E-02	-6.3666E-04	-8.0960E-03
1.6799E+01	−0.0080	-5.3721E-03	-1.0779E-02	2.6279E-03	-2.7795E-03

1.7050E+01	−0.1110	-1.0988E-01	-1.1207E-01	1.1247E-03	-1.0715E-03
1.7279E+01	−0.2090	-2.1089E-01	-2.0835E-01	-1.8850E-03	6.5016E-04
1.7489E+01	−0.3030	-3.0616E-01	-2.9701E-01	-3.1616E-03	5.9866E-03

Table S30. PWP201 photovoltaic panel parameters and range for 15 populations

Algorithm	I_{ph} [A]	I_{o1} [A]	n_1	R_s [Ω]	R_{sh} [Ω]	I_{o2} [A]	n_2
Range Set SDM	0–2	E-12 – E-05	1-2*36	0-2	0-1000		
BMOA SDM	1.0335E+00	7.9587E-06	5.2062E+01	1.0915E+00	1.0000E+03		
Range Set DDM	0–2	E-12 – E-05	1-2*36	0-2	0-1000	E-12 – E-05	1-2*36
BMOA DDM	1.0350E+00	1.0000E-05	7.2000E+01	1.3013E+00	5.4618E+02	9.2563E-07	4.4188E+01

Table S31. (I, V) points of Sharp ND-R250A5 photovoltaic panel for 500 epochs.

Measured data		SDM-BMOA	DDM-BMOA	Error Values	
V [V]	I [A]	I_c [A]	I_c [A]	SDM	DDM
0.0000E+00	9.1500E+00	9.2218E+00	9.0222E+00	7.1833E-02	-1.2782E-01
7.7100E+00	9.1400E+00	9.2187E+00	9.0079E+00	7.8656E-02	-1.3210E-01
1.0980E+01	9.1200E+00	9.2128E+00	9.0015E+00	9.2849E-02	-1.1849E-01
1.4550E+01	9.1100E+00	9.1885E+00	8.9915E+00	7.8487E-02	-1.1848E-01
1.6360E+01	9.1000E+00	9.1540E+00	8.9804E+00	5.4022E-02	-1.1955E-01
1.8000E+01	9.0700E+00	9.0915E+00	8.9579E+00	2.1542E-02	-1.1214E-01
1.9150E+01	9.0200E+00	9.0161E+00	8.9253E+00	-3.8747E-03	-9.4732E-02
2.0040E+01	8.9500E+00	8.9304E+00	8.8821E+00	-1.9635E-02	-6.7926E-02
2.0870E+01	8.8600E+00	8.8202E+00	8.8185E+00	-3.9800E-02	-4.1508E-02
2.1670E+01	8.7300E+00	8.6796E+00	8.7266E+00	-5.0434E-02	-3.4065E-03
2.2360E+01	8.5800E+00	8.5248E+00	8.6141E+00	-5.5157E-02	3.4068E-02
2.3020E+01	8.4000E+00	8.3426E+00	8.4689E+00	-5.7435E-02	6.8926E-02
2.3620E+01	8.2000E+00	8.1443E+00	8.2985E+00	-5.5717E-02	9.8520E-02
2.4150E+01	8.0000E+00	7.9387E+00	8.1100E+00	-6.1268E-02	1.1003E-01

2.4610E+01	7.8000E+00	7.7374E+00	7.9163E+00	-6.2622E-02	1.1632E-01
2.5020E+01	7.6000E+00	7.5394E+00	7.7188E+00	-6.0556E-02	1.1884E-01
2.5390E+01	7.4000E+00	7.3463E+00	7.5209E+00	-5.3695E-02	1.2093E-01
2.5750E+01	7.2000E+00	7.1396E+00	7.3023E+00	-6.0433E-02	1.0234E-01
2.6380E+01	6.8000E+00	6.7488E+00	6.8808E+00	-5.1206E-02	8.0809E-02
2.6940E+01	6.4000E+00	6.3683E+00	6.4634E+00	-3.1667E-02	6.3412E-02
2.7460E+01	6.0000E+00	5.9833E+00	6.0359E+00	-1.6745E-02	3.5878E-02
2.7940E+01	5.6000E+00	5.6064E+00	5.6188E+00	6.4368E-03	1.8842E-02
2.8400E+01	5.2000E+00	5.2190E+00	5.1893E+00	1.8966E-02	-1.0682E-02
2.8840E+01	4.8000E+00	4.8264E+00	4.7568E+00	2.6430E-02	-4.3204E-02
2.9250E+01	4.4000E+00	4.4549E+00	4.3603E+00	5.4880E-02	-3.9686E-02
2.9660E+01	4.0000E+00	4.0519E+00	3.9266E+00	5.1894E-02	-7.3392E-02
3.0050E+01	3.6000E+00	3.6610E+00	3.5193E+00	6.1007E-02	-8.0734E-02
3.0440E+01	3.2000E+00	3.2405E+00	3.0791E+00	4.0543E-02	-1.2091E-01
3.0810E+01	2.8000E+00	2.8413E+00	2.6808E+00	4.1273E-02	-1.1920E-01
3.1170E+01	2.4000E+00	2.4434E+00	2.2966E+00	4.3433E-02	-1.0344E-01
3.1520E+01	2.0000E+00	2.0505E+00	1.9320E+00	5.0502E-02	-6.7961E-02
3.1880E+01	1.6000E+00	1.6033E+00	1.5021E+00	3.3234E-03	-9.7884E-02
3.2220E+01	1.2000E+00	1.1949E+00	1.1423E+00	-5.0585E-03	-5.7748E-02
3.2550E+01	8.0000E-01	7.9958E-01	8.1507E-01	-4.2399E-04	1.5069E-02
3.2890E+01	4.0000E-01	3.4809E-01	4.2213E-01	-5.1914E-02	2.2135E-02
3.3220E+01	0.0000E+00	-8.9013E-02	6.4884E-02	-8.9013E-02	6.4884E-02

Table S232. Sharp ND-R250A5 photovoltaic panel parameters and range for 500 epochs

Algorithm	I_{ph} [A]	I_{o1} [A]	n_1	R_s [Ω]	R_{sh} [Ω]	I_{o2} [A]	n_2
Range Set SDM	0-10	E-12 – E-5	1-2*60	0-1	0-5500		
BMOA SDM	9.2228E+00	9.9109E-06	8.4394E+01	5.3448E-01	5.3876E+03		

Range Set DDM	0-10	E-12 – E-5	1-2*36	0-1	0-1000	E-12 – E-5	1-2*36
BMOA DDM	9.0333E+00	1.0000E-12	5.1116E+01	6.5754E-01	5.4179E+02	1.3700E-08	5.7196E+01

Table S33. (I, V) points of Sharp ND-R250A5 photovoltaic panel for 30 populations.

**Measured data**		**SDM-BMOA**	**DDM-BMOA**	**Error Values**	
**V [V]**	**I [A]**	**I_c [A]**	**I_c [A]**	**SDM**	**DDM**
0.0000E+00	9.1500E+00	9.2225E+00	9.3489E+00	7.2478E-02	1.9892E-01
7.7100E+00	9.1400E+00	9.2194E+00	9.1128E+00	7.9386E-02	-2.7193E-02
1.0980E+01	9.1200E+00	9.2137E+00	9.0130E+00	9.3719E-02	-1.0699E-01
1.4550E+01	9.1100E+00	9.1898E+00	8.9036E+00	7.9780E-02	-2.0639E-01
1.6360E+01	9.1000E+00	9.1557E+00	8.8476E+00	5.5748E-02	-2.5243E-01
1.8000E+01	9.0700E+00	9.0938E+00	8.7949E+00	2.3839E-02	-2.7513E-01
1.9150E+01	9.0200E+00	9.0189E+00	8.7539E+00	-1.0882E-03	-2.6608E-01
2.0040E+01	8.9500E+00	8.9335E+00	8.7162E+00	-1.6451E-02	-2.3376E-01
2.0870E+01	8.8600E+00	8.8237E+00	8.6708E+00	-3.6272E-02	-1.8923E-01
2.1670E+01	8.7300E+00	8.6834E+00	8.6103E+00	-4.6648E-02	-1.1968E-01
2.2360E+01	8.5800E+00	8.5287E+00	8.5364E+00	-5.1252E-02	-4.3637E-02
2.3020E+01	8.4000E+00	8.3465E+00	8.4368E+00	-5.3548E-02	3.6765E-02
2.3620E+01	8.2000E+00	8.1480E+00	8.3124E+00	-5.1984E-02	1.1245E-01
2.4150E+01	8.0000E+00	7.9422E+00	8.1656E+00	-5.7813E-02	1.6559E-01
2.4610E+01	7.8000E+00	7.7405E+00	8.0057E+00	-5.9513E-02	2.0571E-01
2.5020E+01	7.6000E+00	7.5422E+00	7.8345E+00	-5.7834E-02	2.3454E-01
2.5390E+01	7.4000E+00	7.3486E+00	7.6560E+00	-5.1377E-02	2.5598E-01
2.5750E+01	7.2000E+00	7.1414E+00	7.4503E+00	-5.8600E-02	2.5027E-01
2.6380E+01	6.8000E+00	6.7497E+00	7.0371E+00	-5.0276E-02	2.3708E-01
2.6940E+01	6.4000E+00	6.3684E+00	6.6114E+00	-3.1577E-02	2.1137E-01
2.7460E+01	6.0000E+00	5.9825E+00	6.1619E+00	-1.7461E-02	1.6188E-01
2.7940E+01	5.6000E+00	5.6050E+00	5.7172E+00	5.0463E-03	1.1725E-01

2.8400E+01	5.2000E+00	5.2170E+00	5.2527E+00	1.6953E-02	5.2684E-02
2.8840E+01	4.8000E+00	4.8239E+00	4.7823E+00	2.3898E-02	-1.7693E-02
2.9250E+01	4.4000E+00	4.4521E+00	4.3604E+00	5.2095E-02	-3.9614E-02
2.9660E+01	4.0000E+00	4.0488E+00	3.8921E+00	4.8836E-02	-1.0785E-01
3.0050E+01	3.6000E+00	3.6579E+00	3.4640E+00	5.7920E-02	-1.3598E-01
3.0440E+01	3.2000E+00	3.2374E+00	2.9971E+00	3.7444E-02	-2.0288E-01
3.0810E+01	2.8000E+00	2.8385E+00	2.5949E+00	3.8477E-02	-2.0510E-01
3.1170E+01	2.4000E+00	2.4412E+00	2.2205E+00	4.1152E-02	-1.7955E-01
3.1520E+01	2.0000E+00	2.0490E+00	1.8823E+00	4.8971E-02	-1.1772E-01
3.1880E+01	1.6000E+00	1.6025E+00	1.4635E+00	2.4768E-03	-1.3653E-01
3.2220E+01	1.2000E+00	1.1952E+00	1.1513E+00	-4.7882E-03	-4.8724E-02
3.2550E+01	8.0000E-01	8.0126E-01	8.9377E-01	1.2585E-03	9.3769E-02
3.2890E+01	4.0000E-01	3.5115E-01	5.5666E-01	-4.8852E-02	1.5666E-01
3.3220E+01	0.0000E+00	-8.4251E-02	2.7861E-01	-8.4251E-02	2.7861E-01

Table S34. Sharp ND-R250A5 photovoltaic panel parameters and range for 30 populations

Algorithm	I_{ph} [A]	I_{o1} [A]	n_1	R_s [Ω]	R_{sh} [Ω]	I_{o2} [A]	n_2
Range Set SDM	0-10	E-12 – E-5	1-2*60	0-1	0-5500		
BMOA SDM	9.2234E+00	9.2484E-06	8.3974E+01	5.3695E-01	5.5000E+03		
Range Set DDM	0-10	E-12 – E-5	1-2*36	0-1	0-1000	E-12 – E-5	1-2*36
BMOA DDM	9.5533E+00	1.0000E-12	3.9149E+01	7.2876E-01	3.2623E+01	1.0000E-12	4.2439E+01

Table 35. (I, V) points of Sharp ND-R250A5 photovoltaic panel for 15 populations.

Measured data		SDM-BMOA	DDM-BMOA	Error Values	
V [V]	I [A]	I_c [A]	I_c [A]	SDM	DDM
0.0000E+00	9.1500E+00	9.1883E+00	9.1631E+00	3.8340E-02	1.3063E-02

7.7100E+00	9.1400E+00	9.1859E+00	9.1496E+00	4.5868E-02	9.6317E-03
1.0980E+01	9.1200E+00	9.1817E+00	9.1421E+00	6.1693E-02	2.2123E-02
1.4550E+01	9.1100E+00	9.1630E+00	9.1242E+00	5.2988E-02	1.4195E-02
1.6360E+01	9.1000E+00	9.1348E+00	9.1008E+00	3.4820E-02	8.2765E-04
1.8000E+01	9.0700E+00	9.0813E+00	9.0564E+00	1.1289E-02	-1.3644E-02
1.9150E+01	9.0200E+00	9.0142E+00	8.9991E+00	-5.7503E-03	-2.0870E-02
2.0040E+01	8.9500E+00	8.9360E+00	8.9305E+00	-1.4009E-02	-1.9529E-02
2.0870E+01	8.8600E+00	8.8333E+00	8.8380E+00	-2.6738E-02	-2.2019E-02
2.1670E+01	8.7300E+00	8.6996E+00	8.7147E+00	-3.0371E-02	-1.5276E-02
2.2360E+01	8.5800E+00	8.5503E+00	8.5740E+00	-2.9728E-02	-5.9883E-03
2.3020E+01	8.4000E+00	8.3720E+00	8.4030E+00	-2.7993E-02	2.9927E-03
2.3620E+01	8.2000E+00	8.1760E+00	8.2121E+00	-2.3992E-02	1.2064E-02
2.4150E+01	8.0000E+00	7.9710E+00	8.0097E+00	-2.9026E-02	9.6968E-03
2.4610E+01	7.8000E+00	7.7689E+00	7.8083E+00	-3.1146E-02	8.2952E-03
2.5020E+01	7.6000E+00	7.5692E+00	7.6080E+00	-3.0751E-02	7.9733E-03
2.5390E+01	7.4000E+00	7.3739E+00	7.4109E+00	-2.6129E-02	1.0890E-02
2.5750E+01	7.2000E+00	7.1638E+00	7.1976E+00	-3.6166E-02	-2.3514E-03
2.6380E+01	6.8000E+00	6.7662E+00	6.7926E+00	-3.3809E-02	-7.4346E-03
2.6940E+01	6.4000E+00	6.3787E+00	6.3967E+00	-2.1346E-02	-3.2878E-03
2.7460E+01	6.0000E+00	5.9862E+00	5.9951E+00	-1.3817E-02	-4.9329E-03
2.7940E+01	5.6000E+00	5.6028E+00	5.6033E+00	2.8326E-03	3.3263E-03
2.8400E+01	5.2000E+00	5.2089E+00	5.2008E+00	8.9061E-03	8.2961E-04
2.8840E+01	4.8000E+00	4.8106E+00	4.7946E+00	1.0570E-02	-5.4453E-03
2.9250E+01	4.4000E+00	4.4358E+00	4.4152E+00	3.5838E-02	1.5250E-02
2.9660E+01	4.0000E+00	4.0290E+00	4.0027E+00	2.9000E-02	2.7261E-03
3.0050E+01	3.6000E+00	3.6367E+00	3.6078E+00	3.6666E-02	7.8443E-03
3.0440E+01	3.2000E+00	3.2145E+00	3.1825E+00	1.4476E-02	-1.7479E-02

3.0810E+01	2.8000E+00	2.8168E+00	2.7862E+00	1.6811E-02	-1.3811E-02
3.1170E+01	2.4000E+00	2.4227E+00	2.3961E+00	2.2672E-02	-3.8678E-03
3.1520E+01	2.0000E+00	2.0359E+00	2.0166E+00	3.5868E-02	1.6628E-02
3.1880E+01	1.6000E+00	1.5934E+00	1.5792E+00	-6.6116E-03	-2.0788E-02
3.2220E+01	1.2000E+00	1.1946E+00	1.1922E+00	-5.3897E-03	-7.8073E-03
3.2550E+01	8.0000E-01	8.1205E-01	8.2567E-01	1.2047E-02	2.5667E-02
3.2890E+01	4.0000E-01	3.7222E-01	3.9999E-01	-2.7778E-02	-7.1367E-06
3.3220E+01	0.0000E+00	-4.9720E-02	-3.1416E-03	-4.9720E-02	-3.1416E-03

Table S36. Sharp ND-R250A5 photovoltaic panel parameters and range for 15 populations

Algorithm	I_{ph} [A]	I_{o1} [A]	n_1	R_s [Ω]	R_{sh} [Ω]	I_{o2} [A]	n_2
Range Set SDM	0-10	E-12 – E-5	1-2*60	0-1	0-5500		
BMOA SDM	9.1893E+00	3.7562E-06	7.8874E+01	5.6101E-01	5.5000E+03		
Range Set DDM	0-10	E-12 – E-5	1-2*36	0-1	0-1000	E-12 – E-5	1-2*36
BMOA DDM	9.1722E+00	9.1041E-07	7.2000E+01	5.9216E-01	5.9659E+02	1.0000E-12	7.2000E+01

Table S37. (I, V) points of Kyocera KC200GT photovoltaic panel for 500 epochs.

Measured data		SDM-BMOA	DDM-BMOA	Error Values	
V [V]	I [A]	I_c [A]	I_c [A]	SDM	DDM
4.0900E-02	8.1761E+00	8.1445E+00	8.1464E+00	-3.1625E-02	-2.9677E-02
1.0472E+00	8.1761E+00	8.1425E+00	8.1441E+00	-3.3643E-02	-3.2024E-02
2.0534E+00	8.1761E+00	8.1404E+00	8.1417E+00	-3.5663E-02	-3.4372E-02
3.0707E+00	8.1364E+00	8.1384E+00	8.1394E+00	1.9951E-03	2.9516E-03
4.0769E+00	8.1364E+00	8.1364E+00	8.1370E+00	-3.4648E-05	5.9246E-04
4.9082E+00	8.1364E+00	8.1347E+00	8.1350E+00	-1.7183E-03	-1.3635E-03
6.0129E+00	8.1178E+00	8.1324E+00	8.1324E+00	1.4632E-02	1.4624E-02
7.0192E+00	8.1178E+00	8.1304E+00	8.1300E+00	1.2560E-02	1.2221E-02

8.0255E+00	8.1178E+00	8.1283E+00	8.1276E+00	1.0456E-02	9.7866E-03
9.0427E+00	8.0781E+00	8.1261E+00	8.1251E+00	4.7985E-02	4.6979E-02
1.0049E+01	8.0781E+00	8.1239E+00	8.1225E+00	4.5761E-02	4.4422E-02
1.1066E+01	8.0781E+00	8.1215E+00	8.1198E+00	4.3398E-02	4.1722E-02
1.1974E+01	8.0781E+00	8.1192E+00	8.1173E+00	4.1144E-02	3.9167E-02
1.2991E+01	8.0595E+00	8.1165E+00	8.1142E+00	5.6976E-02	5.4660E-02
1.3998E+01	8.0595E+00	8.1134E+00	8.1107E+00	5.3850E-02	5.1201E-02
1.5015E+01	8.0385E+00	8.1096E+00	8.1066E+00	7.1099E-02	6.8116E-02
1.6021E+01	8.0385E+00	8.1050E+00	8.1017E+00	6.6485E-02	6.3181E-02
1.7027E+01	8.0199E+00	8.0990E+00	8.0954E+00	7.9110E-02	7.5501E-02
1.8045E+01	8.0199E+00	8.0908E+00	8.0870E+00	7.0942E-02	6.7061E-02
1.9051E+01	8.0012E+00	8.0796E+00	8.0755E+00	7.8367E-02	7.4275E-02
2.0068E+01	7.9802E+00	8.0632E+00	8.0590E+00	8.3020E-02	7.8819E-02
2.1074E+01	7.9802E+00	8.0395E+00	8.0354E+00	5.9341E-02	5.5225E-02
2.2081E+01	7.9616E+00	8.0045E+00	8.0008E+00	4.2913E-02	3.9184E-02
2.2999E+01	7.9219E+00	7.9577E+00	7.9547E+00	3.5761E-02	3.2809E-02
2.4017E+01	7.9219E+00	7.8806E+00	7.8793E+00	-4.1321E-02	-4.2562E-02
2.5023E+01	7.8823E+00	7.7653E+00	7.7671E+00	-1.1696E-01	-1.1521E-01
2.6029E+01	7.7844E+00	7.5913E+00	7.5980E+00	-1.9310E-01	-1.8640E-01
2.7046E+01	7.5699E+00	7.3256E+00	7.3400E+00	-2.4430E-01	-2.2986E-01
2.8053E+01	7.1385E+00	6.9315E+00	6.9565E+00	-2.0699E-01	-1.8195E-01
2.9070E+01	6.4717E+00	6.3365E+00	6.3751E+00	-1.3517E-01	-9.6605E-02
3.0076E+01	5.4155E+00	5.4699E+00	5.5203E+00	5.4366E-02	1.0476E-01
3.1181E+01	3.8091E+00	4.0549E+00	4.1067E+00	2.4582E-01	2.9760E-01
3.2100E+01	2.1443E+00	2.3491E+00	2.3782E+00	2.0484E-01	2.3392E-01
3.2920E+01	4.4000E-01	2.5898E-01	2.3150E-01	-1.8102E-01	-2.0850E-01

Table S38. Kyocera KC200GT photovoltaic parameters and range for 500 epochs

Algorithm	I_{ph} [A]	I_{o1} [A]	n_1	R_s [Ω]	R_{sh} [Ω]	I_{o2} [A]	n_2
Range Set SDM	0-10	E-12 – E-5	1-128	0-0.5	0-500		
BMOA SDM	8.1453E+00	7.7480E-06	9.2739E+01	4.6913E-02	5.0000E+02		
Range Set DDM	0-10	E-12 – E-5	1-128	0-0.5	0-500	E-12 – E-5	1-128
BMOA DDM	8.1471E+00	1.5167E-06	9.9797E+01	3.1730E-02	4.2981E+02	7.2875E-06	9.2804E+01

Table S39. (I, V) points of Kyocera KC200GT photovoltaic panel for 30 populations.

Measured data		SDM-BMOA	DDM-BMOA	Error Values	
V [V]	I [A]	I_c [A]	I_c [A]	SDM	DDM
4.0900E-02	8.1761E+00	8.1416E+00	8.1416E+00	-3.4484E-02	-3.4463E-02
1.0472E+00	8.1761E+00	8.1396E+00	8.1396E+00	-3.6503E-02	-3.6481E-02
2.0534E+00	8.1761E+00	8.1376E+00	8.1376E+00	-3.8524E-02	-3.8503E-02
3.0707E+00	8.1364E+00	8.1355E+00	8.1356E+00	-8.6950E-04	-8.4813E-04
4.0769E+00	8.1364E+00	8.1335E+00	8.1335E+00	-2.9026E-03	-2.8812E-03
4.9082E+00	8.1364E+00	8.1318E+00	8.1318E+00	-4.5901E-03	-4.5687E-03
6.0129E+00	8.1178E+00	8.1296E+00	8.1296E+00	1.1752E-02	1.1774E-02
7.0192E+00	8.1178E+00	8.1275E+00	8.1275E+00	9.6703E-03	9.6916E-03
8.0255E+00	8.1178E+00	8.1254E+00	8.1254E+00	7.5524E-03	7.5737E-03
9.0427E+00	8.0781E+00	8.1232E+00	8.1232E+00	4.5060E-02	4.5081E-02
1.0049E+01	8.0781E+00	8.1209E+00	8.1209E+00	4.2805E-02	4.2826E-02
1.1066E+01	8.0781E+00	8.1185E+00	8.1185E+00	4.0399E-02	4.0420E-02
1.1974E+01	8.0781E+00	8.1162E+00	8.1162E+00	3.8091E-02	3.8113E-02
1.2991E+01	8.0595E+00	8.1133E+00	8.1134E+00	5.3838E-02	5.3859E-02
1.3998E+01	8.0595E+00	8.1101E+00	8.1101E+00	5.0593E-02	5.0615E-02
1.5015E+01	8.0385E+00	8.1062E+00	8.1062E+00	6.7673E-02	6.7694E-02
1.6021E+01	8.0385E+00	8.1013E+00	8.1013E+00	6.2826E-02	6.2847E-02

1.7027E+01	8.0199E+00	8.0950E+00	8.0950E+00	7.5128E-02	7.5149E-02
1.8045E+01	8.0199E+00	8.0864E+00	8.0864E+00	6.6513E-02	6.6535E-02
1.9051E+01	8.0012E+00	8.0745E+00	8.0746E+00	7.3339E-02	7.3360E-02
2.0068E+01	7.9802E+00	8.0574E+00	8.0574E+00	7.7182E-02	7.7203E-02
2.1074E+01	7.9802E+00	8.0327E+00	8.0327E+00	5.2458E-02	5.2479E-02
2.2081E+01	7.9616E+00	7.9963E+00	7.9963E+00	3.4698E-02	3.4719E-02
2.2999E+01	7.9219E+00	7.9480E+00	7.9480E+00	2.6059E-02	2.6080E-02
2.4017E+01	7.9219E+00	7.8690E+00	7.8690E+00	-5.2898E-02	-5.2877E-02
2.5023E+01	7.8823E+00	7.7518E+00	7.7518E+00	-1.3052E-01	-1.3049E-01
2.6029E+01	7.7844E+00	7.5760E+00	7.5760E+00	-2.0845E-01	-2.0843E-01
2.7046E+01	7.5699E+00	7.3092E+00	7.3092E+00	-2.6074E-01	-2.6072E-01
2.8053E+01	7.1385E+00	6.9152E+00	6.9153E+00	-2.2325E-01	-2.2324E-01
2.9070E+01	6.4717E+00	6.3227E+00	6.3227E+00	-1.4903E-01	-1.4902E-01
3.0076E+01	5.4155E+00	5.4602E+00	5.4602E+00	4.4702E-02	4.4711E-02
3.1181E+01	3.8091E+00	4.0509E+00	4.0509E+00	2.4175E-01	2.4176E-01
3.2100E+01	2.1443E+00	2.3483E+00	2.3483E+00	2.0395E-01	2.0396E-01
3.2920E+01	4.4000E-01	2.5672E-01	2.5673E-01	-1.8328E-01	-1.8327E-01

Table S40. Kyocera KC200GT photovoltaic parameters and range for 30 populations

Algorithm	I_{ph} [A]	I_{o1} [A]	n_1	R_s [Ω]	R_{sh} [Ω]	I_{o2} [A]	n_2
Range Set SDM	0-10	E-12 – E-5	1-128	0-0.5	0-500		
BMOA SDM	8.1423E+00	1.0000E-05	9.4473E+01	3.8411E-02	5.0000E+02		
Range Set DDM	0-10	E-12 – E-5	1-128	0-0.5	0-500	E-12 – E-5	1-128
BMOA DDM	8.1423E+00	1.0000E-12	6.3929E+01	3.8423E-02	5.0000E+02	9.9998E-06	9.4473E+01

Table S41. (I, V) points of Kyocera KC200GT photovoltaic panel for 15 populations.

Measured data		SDM-BMOA	DDM-BMOA	Error Values	
V [V]	I [A]	I_c [A]	I_c [A]	SDM	DDM

4.0900E-02	8.1761E+00	8.1417E+00	8.1395E+00	-3.4445E-02	-3.6577E-02
1.0472E+00	8.1761E+00	8.1396E+00	8.1375E+00	-3.6464E-02	-3.8594E-02
2.0534E+00	8.1761E+00	8.1376E+00	8.1355E+00	-3.8485E-02	-4.0614E-02
3.0707E+00	8.1364E+00	8.1356E+00	8.1334E+00	-8.2917E-04	-2.9559E-03
4.0769E+00	8.1364E+00	8.1335E+00	8.1314E+00	-2.8615E-03	-4.9855E-03
4.9082E+00	8.1364E+00	8.1319E+00	8.1297E+00	-4.5482E-03	-6.6690E-03
6.0129E+00	8.1178E+00	8.1296E+00	8.1275E+00	1.1796E-02	9.6816E-03
7.0192E+00	8.1178E+00	8.1275E+00	8.1254E+00	9.7160E-03	7.6096E-03
8.0255E+00	8.1178E+00	8.1254E+00	8.1233E+00	7.6012E-03	5.5064E-03
9.0427E+00	8.0781E+00	8.1232E+00	8.1211E+00	4.5113E-02	4.3036E-02
1.0049E+01	8.0781E+00	8.1210E+00	8.1189E+00	4.2865E-02	4.0812E-02
1.1066E+01	8.0781E+00	8.1186E+00	8.1165E+00	4.0468E-02	3.8450E-02
1.1974E+01	8.0781E+00	8.1163E+00	8.1143E+00	3.8171E-02	3.6197E-02
1.2991E+01	8.0595E+00	8.1134E+00	8.1115E+00	5.3935E-02	5.2029E-02
1.3998E+01	8.0595E+00	8.1102E+00	8.1084E+00	5.0714E-02	4.8903E-02
1.5015E+01	8.0385E+00	8.1063E+00	8.1047E+00	6.7828E-02	6.6152E-02
1.6021E+01	8.0385E+00	8.1015E+00	8.1000E+00	6.3026E-02	6.1535E-02
1.7027E+01	8.0199E+00	8.0953E+00	8.0941E+00	7.5390E-02	7.4156E-02
1.8045E+01	8.0199E+00	8.0868E+00	8.0859E+00	6.6860E-02	6.5976E-02
1.9051E+01	8.0012E+00	8.0750E+00	8.0746E+00	7.3795E-02	7.3380E-02
2.0068E+01	7.9802E+00	8.0580E+00	8.0582E+00	7.7781E-02	7.7992E-02
2.1074E+01	7.9802E+00	8.0334E+00	8.0344E+00	5.3234E-02	5.4239E-02
2.2081E+01	7.9616E+00	7.9973E+00	7.9993E+00	3.5685E-02	3.7682E-02
2.2999E+01	7.9219E+00	7.9492E+00	7.9522E+00	2.7263E-02	3.0337E-02
2.4017E+01	7.9219E+00	7.8704E+00	7.8748E+00	-5.1470E-02	-4.7115E-02
2.5023E+01	7.8823E+00	7.7534E+00	7.7589E+00	-1.2893E-01	-1.2336E-01
2.6029E+01	7.7844E+00	7.5775E+00	7.5839E+00	-2.0687E-01	-2.0045E-01

2.7046E+01	7.5699E+00	7.3104E+00	7.3168E+00	-2.5947E-01	-2.5310E-01
2.8053E+01	7.1385E+00	6.9158E+00	6.9208E+00	-2.2267E-01	-2.1768E-01
2.9070E+01	6.4717E+00	6.3221E+00	6.3236E+00	-1.4962E-01	-1.4813E-01
3.0076E+01	5.4155E+00	5.4586E+00	5.4554E+00	4.3065E-02	3.9936E-02
3.1181E+01	3.8091E+00	4.0495E+00	4.0421E+00	2.4036E-01	2.3297E-01
3.2100E+01	2.1443E+00	2.3501E+00	2.3435E+00	2.0580E-01	1.9921E-01
3.2920E+01	4.4000E-01	2.6619E-01	2.6832E-01	-1.7381E-01	-1.7168E-01

Table S42. Kyocera KC200GT photovoltaic parameters and range for 15 populations

Algorithm	I_{ph} [A]	I_{o1} [A]	n_1	R_s [Ω]	R_{sh} [Ω]	I_{o2} [A]	n_2
Range Set SDM	0-10	E-12 – E-5	1-128	0-0.5	0-500		
BMOA SDM	8.1424E+00	9.4450E-06	9.4089E+01	4.1473E-02	5.0000E+02		
Range Set DDM	0-10	E-12 – E-5	1-128	0-0.5	0-500	E-12 – E-5	1-128
BMOA DDM	8.1404E+00	7.5947E-06	9.2621E+01	5.0185E-02	5.0000E+02	1.0000E-12	1.2800E+02
